# Unique immunologic patterns in fibromyalgia

**DOI:** 10.1186/1472-6890-12-25

**Published:** 2012-12-17

**Authors:** Frederick G Behm, Igor M Gavin, Oleksiy Karpenko, Valerie Lindgren, Sujata Gaitonde, Peter A Gashkoff, Bruce S Gillis

**Affiliations:** 1 Department of Pathology, University of Illinois at Chicago (UIC), Chicago, IL, USA; 2 Research Resource Center, University of Illinois at Chicago, Chicago, IL, USA

## Abstract

**Background:**

Fibromyalgia (FM) is a clinical syndrome characterized by chronic pain and allodynia. The diagnosis of FM has been one of exclusion as a test to confirm the diagnosis is lacking. Recent data highlight the role of the immune system in FM. Aberrant expressions of immune mediators, such as cytokines, have been linked to the pathogenesis and traits of FM. We therefore determined whether cytokine production by immune cells is altered in FM patients by comparing the cellular responses to mitogenic activators of stimulated blood mononuclear cells of a large number of patients with FM to those of healthy matched individuals.

**Methods:**

Plasma and peripheral blood mononuclear cells (PBMC) were collected from 110 patients with the clinical diagnosis of FM and 91 healthy donors. Parallel samples of PBMC were cultured overnight in medium alone or in the presence of mitogenic activators; PHA or PMA in combination with ionomycin. The cytokine concentrations of IFN-γ, IL-5, IL-6, IL-8, IL-10, MIP-1β , MCP-1, and MIP1-α in plasma as well as in cultured supernatants were determined using a multiplex immunoassay using bead array technology.

**Results:**

Cytokine levels of stimulated PBMC cultures of healthy control subjects were significantly increased as compared to matched non-stimulated PBMC cultures. In contrast, the concentrations of most cytokines were lower in stimulated samples from patients with FM compared to controls. The decreases of cytokine concentrations in patients samples ranged from 1.5-fold for MIP-1β to 10.2-fold for IL-6 in PHA challenges. In PMA challenges, we observed 1.8 to 4-fold decreases in the concentrations of cytokines in patient samples.

**Conclusion:**

The cytokine responses to mitogenic activators of PBMC isolated from patients with FM were significantly lower than those of healthy individuals, implying that cell-mediated immunity is impaired in FM patients. This novel cytokine assay reveals unique and valuable immunologic traits, which, when combined with clinical patterns, can offer a diagnostic methodology in FM.

## Background

Fibromyalgia (FM) is a chronic pain and fatigue syndrome that affects ten to fifteen million adults in the United States. Patients with FM display diffuse hyperalgesia (i.e., increased response to painful stimuli) and/or allodynia (i.e., a heightened sensitivity to stimuli that are not normally painful). Other clinical manifestations include debilitating fatigue, sleep disturbance, joint stiffness, cognitive dysfunction and depression. In the past, this syndrome has been known as fibrositis, nonarticular rheumatism, the chronic fatigue syndrome, myodysneuria, fibromyositis and muscular rheumatism, among others. The diagnosis of FM is one of exclusion as there are no specific laboratory tests or radiographic/imaging studies to confirm this diagnosis. The only physical finding currently used for diagnosis is excess tenderness on palpation of soft tissues [1-5]. Patients who have been labeled with the diagnosis of FM commonly spend years seeking confirmation of this health problem. The lack of an objective test to either confirm or rule-out the existence of FM not infrequently results in some patients being labeled as neurotic, hypochondrical or emotionally unstable.

The etiology of FM is not completely understood but the current hypothesis is that FM arises from interactions between the autonomic central nervous system, the hypothalamic-pituitary-adrenal axis and the immune system [6-9]. In the past, FM was considered a rheumatologic disorder based upon the assumption that it is a type of inflammatory fibro-connective malady. This is not surprising given that FM is common in patients with autoimmune disorders, such as systemic lupus erythematosus [10,11], Sjogren's Syndrome [12,13], and rheumatoid arthritis [14]. Recent studies have highlighted the role of the immune system in the pathogenesis of this disease [15]. As early as 1988, it was noted that aberrant expressions of immune mediators such as cytokines may contribute to the onset of disease symptoms [16]. However, reports on changes in serum cytokine levels in FM patients have revealed conflicting results [17,18]. To uncover direct evidence of the imbalance in cytokine production and secretion in FM patients we evaluated the cytokine-producing activity of immune cells in FM patients. We measured plasma cytokine levels in a group of 110 patients with a diagnosis of FM and we determined responses to mitogen challenges of their peripheral blood mononuclear cells (PBMC). The cytokine levels of these patients were compared with those in a group of 91 matched healthy controls.

## Methods

### Study groups

This study was approved by the University of Illinois at Chicago, College of Medicine Institutional Review Board and all participants consented to participate in this study. The study was performed in a blinded manner. A total of 110 fibromyalgia patients and 91 control subjects were recruited. Patient characteristics are shown in Table 1. All patients had fibromyalgia symptoms and had been diagnosed with fibromyalgia for at least one year. Patients underwent two separate and independent physical examinations to confirm that they met the criteria according to the current standards of the American College of Rheumatology [1]. All fibromyalgia patients had been off FDA-approved fibromyalgia drugs (milnacipran, pregabalin, duloxetine) for at least two weeks. Patients with any other potentially confounding variables were excluded, i.e., if they had any other rheumatologic disorders, any autoimmune or immunologic diseases, any inflammatory disorder, any infectious disorder or any neoplastic disorder or were receiving anti-cancer treatment or had been taking medications affecting the immune system, including any anti-inflammatory medications. Matched control subjects lacked a history of any type of chronic or acute illnesses and none were using any medications, OTC or prescription drugs that had any known immunologic effects. They ranged in age from 18 years to 76 years. The median age was 39 years, and the average age was 40 years. Of the 91 control subjects, 87 were Caucasian.

**Table 1:** Characteristics of patients

**Cohort size**	**10**
Age	
Range	18-82
Median	53.2
Mean	52.2
Sex	
Female	98
Male	12
Ethnicity	
Caucasian*	101
Other	9
Years of living with FM	
Range	1-46
Median	30
Mean	16
Mode	10
Fibromyalgia Symptoms/Co-Morbidities	
Tender Muscle Points	101
Nonrestorative Sleep/Insomnia	89
Chronic Fatigue	99
Mental Fogginess	93
Psychotherapist Confirmed Diagnosis of Depression	9

* The Caucasian classification was also used if patients identified themselves as Hispanic.

### Isolation of PBMC

Twenty eight (28) mL of blood were obtained from patients and healthy volunteers after obtaining their consent for research. Blood was withdrawn in four 7 mL tubes containing 0.081 mL of 15% K_3_ EDTA solution (BD Vacutainer®, BD, Franklin Lakes, NJ) by venipuncture. The samples were coded and shipped to the processing lab in insulated containers at room temperature. Once received, the blood was transferred to a 50ml tube, mixed, split in two 50 ml tubes and diluted 1:1 in GIBCO® Hank’s balanced salt solution (HBSS, Invitrogen, Carlsbad, CA). The resulting suspension was layered on top of 15 ml Ficoll (Histopaque®-1077, Sigma-Aldrich, St. Louis, MO) in 50 mL tubes and centrifuged at 700g for 20 minutes. The layer containing PMBC was harvested and transferred into a new 50 mL tube. The tube was filled with HBSS and the contents were mixed by gentle rocking. Cells were collected by centrifugation at 850g for 10 minutes and resuspended in 10 mL complete RPMI-1640 medium supplemented with 10% fetal bovine serum, 1% penicillin-streptomycin mixture and 1% L-glutamine solution (Invitrogen, Carlsbad, CA).

### PBMC culture

Cells were suspended at a concentration of 10 [6] cells/mL in a supplemented complete RPMI medium. A 3 mL suspension was placed in six-well tissue culture plates (Nalge Nunc International, Rochester, NY) and either 10 μg/mL PHA-P (Sigma-Aldrich®, St. Louis, MO) or a mixture of 1 μg/mL phorbol-12-myristate-13-acetate (PMA) and 1 μg/mL ionomycin (EMD Biosciences, Inc, Darmstadt, Germany) was added to the wells in triplicate. No chemicals were added to three control plates which were used to determine the basal levels of expression. Plates were incubated in a CO_2_ water jacketed incubator (Forma Scientific, Marjetta, OH) for 18 hours.

### Cytokine assay

After overnight culture of PBMC in medium alone or in the presence of mitogens, 1 mL supernatant was removed from each plate, centrifuged at 16,000g at +4°C, for 10 minutes and clear cell-free culture samples were collected, placed in a new 1.7 mL tube and frozen at -80°C. Cytokine and chemokine concentrations in plasma as well as in culture supernatants were determined using a multiplex immunoassay based on the Luminex xMAP™ bead array technology. A custom panel of antibody-conjugated beads for measuring eight human cytokines (IL-5, IL-6, IL-8, IFN-γ, IL-10, MIP-1α, MIP-1β, MCP-1) (BioRad Laboratories, Hercules, CA) was used in the assay according to the manufacturer's instructions. Cytokine concentrations were determined in both undiluted and diluted samples. Blank medium was used as a negative control. Serial dilutions of cytokine standards were run in duplicates in each assay; their readings were used for calculating standard curves. In addition, pooled culture supernatants obtained from activated cells served as a positive control. Fluorescence was measured with a Bio-Plex 200 fluorescence bead reader (BioRad Laboratories, Hercules, CA).

### Statistical analysis

Fluorescence intensities were transferred into R [19] for converting to concentration values. The standard curves were fitted using the 4-parameter logistic (4-PL) or, in cases when the 4-PL model failed, the 5-parameter logistic (5-PL) model [20]. In total, 192 (24 plates times 8 cytokines) standard curves were fitted. The concentrations of cytokines in the plasma of controls and FM patients were compared using an unpaired two-sided *t*-test with unequal variance. This same test was used to compare the concentrations of cytokines in unstimulated and stimulated PBMC cultures of controls. The variances and means of the cytokine concentrations in patients and healthy individuals were compared using the F-test and *t*-test, respectively. The differences in variance were not detected at α=0.1, except for IL-5 in the PHA challenge. The descriptive statistics of the groups as well as the P-values of the latter *t*-test were also calculated. In order to determine whether the cytokine concentrations after PHA or PMA stimulations changed in the same direction for each individual patient, we calculated the pair-wise correlations between the cytokines using Spearman's rank correlation test. For all of the statistical tests, we used the stats package for R [19].

## Results

### Concentrations of cytokines in plasma of FM patients

Concentrations of eight cytokines, IFN-γ, IL-5, IL-6, IL-8, IL-10, MIP-1β, MCP-1, and MIP1-α were measured in the plasma of the 110 FM patients and the 91 control subjects (Table 2). The levels of most cytokines were below the lower limits of detection. When cytokine levels in FM samples were compared with the control values, no statistically significant differences were found at α=0.05.

**Table 2:** Concentrations of cytokines in plasma of FM patients and controls

**Cytokine**	**Controls**			**Patients**			**P-value**
	**N**	**Mean**	**SD**	**N**	**Mean**	**SD**	
MCP-1	65	25	19	95	23	23	0.5681
IL-5	30	24.0	86.0	26	28	103	0.8685
IL-6	39	23	59	47	18	29	0.6511
IL-8	62	22	13	43	25	49	0.7031
IL-10	59	125	713	54	18	41	0.2531
IFN-γ	54	659	1935	51	681	766	0.9383
MIP-1α	36	6	7	24	9	24	0.6807
MIP-1β	87	61	31	107	52	49	0.1506

Concentrations of cytokines in pg/ml are shown.

N, number of subjects whose cytokine values within the detection ranges.

### Levels of cytokines in stimulated PBMC cultures

To determine if cytokine production by immune cells is altered in FM patients, we measured cellular responses to the mitogenic activators, phytohemagglutinin (PHA) and phorbol-12-myristate-13-acetate (PMA) in combination with ionomycin. PHA, a lectin from *Phaseolus vulgaris* (red kidney bean), induces PBMC proliferation as well as the secretion of cytokines [21,22]. PMA and ionomycin also stimulate PBMC and activate extracellular expressions of cytokines [23-25]. We therefore cultured PBMC which were isolated from the blood samples from the 110 patients and the 91 control subjects in the presence of either PHA or PMA/ionomycin and determined the concentrations of eight cytokines in culture supernatants. As shown in Table 3, unstimulated PBMC cultures produced low levels of cytokines in the control subjects. Of these 91 tested samples, about half were below the lower detection limits. In contrast, in both of the PHA and PMA/ionomycin challenges, extracellular expressions of all cytokines were significantly increased, resulting in increased cytokine levels in the culture supernatants. The only exception was the cytokine IL-5 concentrations in the PHA challenge, whose increase was not statistically significant.

**Table 3:** Concentrations of cytokines in stimulated PBMC cultures of controls

**Cytokine**	**Median detection ranges**	**Unstimulated**			**PHA-stimulated**				**PMA/Ionomycin-stimulated**			
		**N**	**Mean**	**SD**	**N**	**Mean**	**SD**	**P-value**	**N**	**Mean**	**SD**	**P-value**
MCP-1	3-14,000	50	299	217	90	3497	4384	<.001	91	1224	1126	<.001
IL-5	1-13,000	16	2.6	1.3	32	48	150	0.10	87	64	64	<.001
IL-6	1-33,000	59	215	380	91	2799	4182	<.001	91	532	474	<.001
IL-8	2-60,000	56	771	659	89	17456	24246	<.001	90	17607	10103	<.001
IL-10	2-63,000	44	23	29	65	80	94	<.001	87	63	60	<.001
IFN-γ	5-224,000	40	538	338	70	1516	2425	0.001	91	36726	55772	<.001
MIP-1α	1-7,000	56	33	73	91	1084	1773	<.001	90	1246	1051	<.001
MIP-1β	5-14,000	57	222	174	83	4413	5228	<.001	80	6389	4915	<.001

Concentrations of cytokines in pg/ml are shown.

N, number of subjects whose cytokine values were within the detection ranges.

### PBMC responses to mitogens in FM patients versus controls

When cytokine concentrations in patient samples were compared to the control values, the concentrations of most cytokines were lower in FM samples (Table 4). The decrease was statistically significant for all cytokines except for IL-5 in the PHA challenge. Besides muscle tenderness, the vast majority of the FM patients suffered from chronic fatigue, sleep disorders as well as mental fogginess, which is consistent with the universally recognized traits that make up the FM syndrome. Therefore, the samples from the subgroup of patients having the same individual traits had a nearly identical change in cytokine values when compared to the whole group (not shown). Nine FM patients had been diagnosed by a licensed psychotherapist with depression. A statistical analysis was performed on the patients excluding those who had a confirmed diagnosis of depression and it showed similar results (not shown), thereby proving that depression by itself had an extremely limited impact on the cytokine profiles in FM patients.

**Table 4:** Cytokine production in response to stimulation in FM patients compared to healthy controls

**Cytokine**	**PHA**													
	**Controls**						**Patients**						**C**_**mean**_**/ P**_**mean**_	**P-value**
	**N**	**Mean**	**SD**	**Min.**	**Max.**	**Med.**	**N**	**Mean**	**SD**	**Min.**	**Max.**	**Med.**		
MCP-1	90	3497	4384	69	>OOR	1823	109	1968	1993	86	>OOR	1310	1.8	0.003
IL-5	32	47.7	150.1	<OOR	853	14.1	48	7	17	<OOR	89	1.5	7	0.136
IL-6	91	2799	4182	1.2	15592	273	110	276	437	3.1	2255	108	10.2	<.001
IL-8	89	17456	24246	161	>OOR	4933	110	5751	6123	135	37981	3144	3	<.001
IL-10	65	80	94	<OOR	352	38	103	12	15	<OOR	84	6.8	6.5	<.001
IFNγ	70	1516	2425	<OOR	16902	670	101	569	1920	<OOR	15318	124	2.7	0.007
MIP-1α	91	1084	1773	1.9	9159	240	110	204	272	5.7	1598	109	5.3	<.001
MIP-1β	83	4413	5228	122	>OOR	2282	109	2900	2417	395	>OOR	2259	1.5	0.016
**Cytokine**	**PMA/Ionomycin**													
	**Controls**						**Patients**						**C**_**mean**_/ **P**_**mean**_	**P- value**
	**N**	**Mean**	**SD**	**Min.**	**Max.**	**Med.**	**N**	**Mean**	**SD**	**Min.**	**Max.**	**Med.**		
MCP-1	91	1224	1126	150	8295	874	108	436	421	<OOR	2363	292	2.8	<.001
IL-5	87	63.8	63.9	<OOR	374.5	42.5	98	36	54	<OOR	441	17	1.8	0.002
IL-6	91	532	474	26	2108	350	108	242	301	<OOR	1509	109	2.2	<.001
IL-8	90	17607	10103	5724	>OOR	13677	109	8756	6416	215	>OOR	7503	2	<.001
IL-10	87	63	60	<OOR	254	40	90	27	37	<OOR	230	11	2.4	<.001
IFNγ	91	36726	55772	205	339591	16130	103	14584	23047	<OOR	144516	5636	2.5	0.001
MIP-1α	90	1246	1051	105	>OOR	987	109	308	294	<OOR	1551	191	4	<.001
MIP-1β	80	6389	4915	254	>OOR	5177	106	3086	4040	9.5	>OOR	1517	2.1	<.001

Concentrations of cytokines in pg/ml are shown.

N, number of subjects whose cytokine values were within the detection ranges.

OOR, out of range.

In order to better understand the dynamics of changes in individual cytokines in patients, we questioned whether expressions of different cytokines changed similarly in the same samples. We calculated pair-wise correlations between the cytokine concentrations using Spearman's rank correlation (Table 5). All of the cytokines showed moderate to strong correlations with each other with a correlation coefficient *ρ* > 0.4 (all correlations were significant at 1% FDR), suggesting that cytokine dynamics after each treatment is similar in individual patients. No negative correlations were observed, which means that no two cytokine expressions changed in opposite directions.

**Table 5:** Spearman correlation coefficients of cytokine expressions in patients

**PHA**	MCP-1	IL-5	IL-6	IL-8	IL-10	IFNγ	MIP-1α	MIP-1β
MCP-1	1	0.52	0.68	0.80	0.69	0.41	0.61	0.71
IL-5		1	0.67	0.68	0.69	0.65	0.76	0.66
IL-6			1	0.82	0.82	0.76	0.90	0.80
IL-8				1	0.74	0.67	0.84	0.71
IL-10					1	0.57	0.85	0.82
IFNγ						1	0.78	0.55
MIP-1α							1	0.82
MIP-1β								1
**PMA**	MCP-1	IL-5	IL-6	IL-8	IL-10	IFNγ	MIP-1α	MIP-1β
MCP-1	1	0.60	0.64	0.77	0.52	0.49	0.67	0.51
IL-5		1	0.74	0.48	0.67	0.72	0.71	0.69
IL-6			1	0.57	0.83	0.83	0.88	0.83
IL-8				1	0.48	0.39	0.63	0.43
IL-10					1	0.86	0.86	0.85
IFNγ						1	0.85	0.93
MIP-1α							1	0.87
MIP-1β								1

## Discussion

We utilized multiple immunologic methods to develop an objective test that offers an adjunct to the current subjective physical findings which are used to make a diagnosis when a patient is suspected of having FM. This test is based on specific abnormalities in the cytokine levels of stimulated peripheral blood mononuclear cells of such patients. Decreased PBMC responses to challenges regarding IFN-γ, IL-5, IL-6, IL-8, IL-10, MIP-1β, MCP-1 and MIP1-α but not IL-5 were statistically significantly associated with this disorder in a large number of patients with FM. Decreased expressions of IL-6, IL-10, and IFN-γ were also observed for purified protein derivative-stimulated PBMC in patients with Sjogren’s syndrome with myalgia [26]. The fibromyalgia-related cytokine patterns which were identified may lend some insight as to why there has been an overlap in the diagnoses of mental depression and chronic pain in these individuals. Chronically depressed patients are characterized by higher pro-inflammatory cytokine blood levels and lower anti-inflammatory cytokine levels in blood, similar to what has been found in patients with chronic pain [27-31]. Decreased IL-10, IL-1α and IFN-γ production by mitogen-stimulated PBMC was observed in depressed patients which correlated with our findings [32,33].

The methodology used in this study differs significantly from previous studies; therefore the findings of this study cannot be directly compared to the results of previous studies of cytokines in patients with FM. For example, many studies examined levels of cytokines in serum or plasma, used different controls or did not ensure that their patients were not receiving treatments for FM [17,18]. Our findings prove that relying on a methodology that solely measures circulating cytokine levels leads to results that are statistically insignificant and such measurements fail to confer any diagnostic values.

There have been several studies which employed isolated peripheral blood cells which were stimulated with different agents and they measured levels of a limited number of cytokines after stimulation [34-37]. The latter studies further differed from the methods used in this study regarding the types of PBMCs used (e.g., whole blood, isolated lymphocytes and monocytes alone or together) and in the methods of measurement (e.g., ELISA, flow cytometry, intracellular cytokine staining, and RT-PCR). Regardless of the methodology used, our study revealed significant differences in cytokine expressions in PBMCs from FM patients versus control cells, which can be utilized as the basis of a clinical diagnostic test for FM.

The common thread in these studies as related to the current study was a reduction or no increase in the levels of cytokines IL-6, IL-8, IL-10 and IFN-γ in patients with chronic pain.

The fibromyalgia syndrome by definition lacks any consistent patterns regarding pain intensity, which is a totally subjective process. Pain duration is a required criteria per the American College of Rheumatology definition for the diagnosis of FM, which we adhered to. In the past, FM was claimed to be a rheumatologic, neurologic or psychiatric disease despite the fact that there were no objective links to any of those pathways. Our findings uncovered evidence that FM is instead an immunologic disorder. They prove that the immunologic basis of FM occurs independently of any subjective features. Hence, this illustrates the very strong clinical value of our test protocol. The fact that individual cytokines exhibited similar dynamics in patient samples reveals that the FM patients are uniform in regard to their cellular immunologic responses.

## Conclusion

We conclude that the aberrant responses of levels of a large number of cytokines of *in vitro* stimulated PBMC using the methodology in this study is significantly correlated with the clinical diagnosis of FM. This methodology provides a useful confirmation of the clinical diagnosis of FM. Further, this method of evaluating cytokines levels in stimulated PBMC may prove useful in analyzing specific responses to therapeutic modalities and medications in order to determine the efficacy of such interventions.

## Competing interests

The authors declare that they have no competing interests.

## Authors’ contributions

BSG conceived of the study. FGB, IMG, BSG, PAG participated in the study design and coordination of the study. IMG, VL, SG developed the methods and organized data collection. IMG, OK performed data and statistical analyses. FGB, IGM, BSG drafted the manuscript. All authors read and approved the final manuscript.

## Pre-publication history

The pre-publication history for this paper can be accessed here:

http://www.biomedcentral.com/1472-6890/12/25/prepub
